# Comparative evaluation of the effect of different cleaning agents on colour and surface roughness of Invisalign clear aligners: a cross-over randomized controlled trial

**DOI:** 10.1186/s12903-025-06928-w

**Published:** 2025-11-04

**Authors:** Banu Kılıç, Şeymanur Canpolat, Merve Öztürk

**Affiliations:** 1 Department of Orthodontics, Faculty of Dentistry, Bezmialem Vakif University, Istanbul, Turkey; 2 Faculty of Dentistry, Bezmialem Vakif University, Istanbul, Turkey; 3 Department of Orthodontics, Institute of Health Sciences, Bezmialem Vakif University, Istanbul, Turkey

**Keywords:** Cleaning agents, Clear aligners, Colour change, Roughness

## Abstract

**Background:**

Recent advancements in orthodontics have led to the widespread use of clear aligners, appreciated for their aesthetic and comfort advantages. However, standardized cleaning instructions remain undefined, and maintaining the surface integrity of aligners is critical for treatment efficacy and patient satisfaction. Although several studies have evaluated the effects of cleaning agents, comprehensive clinical evidence regarding their impact on the colour stability and surface roughness of Invisalign aligners is still lacking. This study aims to address this gap by comparing the effects of five commonly used cleaning agents on these key properties.

**Objectives:**

To assess the influence of various cleaning agents on both the colour and surface roughness of clear aligners.

**Materials and methods:**

This randomized crossover clinical trial included 23 participants with good oral hygiene, each requiring more than 12 aligners. Participants were randomly assigned to use two different cleaning agents in two consecutive treatment phases. The agents tested were: (1) Invisalign Cleaning Crystals (Align Technology, USA), (2) Efferdent Anti-Bacterial Denture Cleanser (Prestige Brands, USA), (3) Sensodyne Rapid Relief toothpaste (GlaxoSmithKline, UK), (4) Signal White Now Whitening Toothpaste (Unilever, USA), and (5) colourant-free clear soap (La Petit Marseillaise, France). Colour measurements (ΔE) were obtained using a spectrophotometer based on the CIE Lab* system, and surface roughness was evaluated with an optical profilometer. Bonferroni-corrected post hoc pairwise comparisons were conducted using Jamovi version 2.6.26.

**Results:**

Colourant-free clear soap caused the least colour change, while the Cleaning Crystals and Whitening Toothpaste showed the highest. Control (unused) aligners had greater surface roughness than cleaned aligners. Among cleaning agents, the Cleaning Crystals group exhibited significantly higher roughness than most others (*p* < 0.05).

**Conclusions:**

Cleaning agents influence the aesthetic and physical properties of aligners to varying degrees. Colourant-free clear soap demonstrated minimal impact on both colour and surface integrity and may be recommended as a safe and inexpensive option for daily aligner care.

**Trial registration:**

ClinicalTrials.gov Identifier: NCT05213650. Registered 17 January 2022. Retrospectively registered. This randomized controlled trial adheres to the CONSORT 2010 guidelines for reporting.

**Supplementary Information:**

The online version contains supplementary material available at 10.1186/s12903-025-06928-w.

## Introduction

### Introduction to clear aligners

In recent years, orthodontics has seen significant advancements that have reshaped patient preferences and treatment expectations. The aesthetic concerns associated with traditional fixed appliances have led to increased demand for more inconspicuous alternatives [1]. Clear aligner systems, first introduced in 1998 with the support of CAD/CAM technology, utilize custom-made thermoplastic materials—typically polyurethane—to offer a removable and transparent solution for orthodontic treatment. These aligners provide enhanced comfort and aesthetic acceptability, especially for adult patients, and are now widely adopted in clinical practice [2].

### Discoloration and wear associated with aligner use

Maintaining aligner hygiene is essential for both oral health and aesthetic preservation. Manufacturers recommend daily cleaning of aligners using lukewarm water with soap or specialized cleaning agents to minimize bacterial accumulation and preserve material properties [3]. Inadequate hygiene practices can lead to the formation of bacterial biofilm, which has been associated with periodontal inflammation, caries development, and halitosis [4]. Despite guidance to consume only water while wearing aligners, patients frequently ingest pigmented beverages, potentially altering the polymer structure and diminishing transparency [5]. Consequently, discoloration and changes in surface roughness of aligners are concerns for both clinicians and patients seeking long-term treatment efficacy and esthetics.

### Cleaning needs and related literature

Intraoral wear exposes aligners to chemical and mechanical stress, resulting in surface changes and transparency loss. Schuster et al. reported the presence of surface abrasions, plaque deposits, and calcified biofilm in stagnation areas of worn aligners [6]. Similarly, Gracco et al. observed microcracks, erosion, delamination, bacterial adhesion, and biofilm formation after only 14 days of use, accompanied by significant visual degradation [7]. While several studies have investigated biofilm removal from clear aligners using various cleaning methods [5, 8–10], few have assessed alterations in the aligner surface through randomized controlled clinical trials [11, 12]. For instance, Charavet et al., Shpack et al., Lombardo et al., and Pardo et al. investigated the efficacy of different cleaning protocols on microbial cleanliness of removable orthodontic appliances, including clear aligners [5, 8–10]. However, these investigations did not extensively assess how such methods impact the physical and aesthetic properties of the aligner materials. Therefore, there remains a gap in the literature for comprehensive clinical studies that not only evaluate biofilm removal but also examine how different cleaning agents affect the surface roughness and colour stability of clear aligners.

### Study objective and hypothesis

This randomized controlled crossover study aims to evaluate the effects of five commercially available cleaning agents on the surface roughness and colour stability of polyurethane-based clear aligners. The null hypothesis of the study is that different cleaning agents do not significantly affect the surface roughness or colour stability of clear aligners.

## Materials and methods

Sample size calculation was performed using GPower software (version 3.1.9.7), based on previously reported data by Alper et al. [13], to achieve 80% power at α = 0.05, determining 45 aligners per group. The study received ethical approval from theBezmialem Vakıf University Institutional Review Board (protocol number 18.10.2021-E.36478).

The aligners used in this study were worn for 14 days to simulate realistic clinical wear conditions. The manufacturer of the clear aligners recommends that each aligner be worn for approximately 7 to 14 days, depending on the individual treatment plan and the orthodontist's guidance. The 14-day period is commonly applied in routine clinical practice, as it provides sufficient exposure to intraoral factors such as saliva, dietary components, and temperature changes, which can influence the aligner's surface properties. Additionally, Lira et al. demonstrated that a 14-day oral exposure period is sufficient to observe meaningful changes in the chemical, physical, and morphological properties of clear aligners [14]. Therefore, this period was chosen to ensure both clinical relevance and comparability with previous studies investigating similar parameters.

### Study population and eligibility criteria

A total of 23 patients were enrolled throughout the course of the study; however, the study initially commenced with 20 participants who were scheduled to undergo treatment with more than 12 clear aligners. The recruitment and follow-up phases spanned from October 2021 to June 2022. Patients were recruited from the orthodontic clinic of Bezmialem Vakıf University, and written informed consent was obtained from all participants.

Inclusion criteria required participants to be in good general and oral health and to require a treatment plan involving at least 12 aligners. The exclusion criteria included: active periodontal disease, presence of dental caries, systemic disease, use of systemic medications, smoking, bruxism, neuropsychological disorders that could interfere with adherence to study protocols, and regular consumption of foods or beverages known to cause pronounced oral discoloration.

Although the study was originally designed to last six months, the duration was extended to nine months due to the exclusion and replacement of participants. Ultimately, 20 subjects completed the study, contributing a total of 240 aligners to the final analysis (Table 1).

**Table 1:** Baseline average age, standard deviations, and gender distribution by groups

**Group**		***Average Age***	**Gender Distribution**	
			**Male**	**Female**
Cleaning Crystals	T1	23.67 ± 0.92	3	1
	T2	22.92 ± 0.83	2	2
Whitening Toothpaste	T1	16.42 ± 10.33	3	1
	T2	13.67 ± 1.17	1	3
Efferdent	T1	18.67 ± 0.83	1	3
	T2	16.17 ± 0.17	2	2
Toothpaste	T1	20.83 ± 0.92	1	3
	T2	21.08 ± 0.58	1	3
Liquid Soap	T1	14.67 ± 3.75	1	3
	T2	24.25 ± 6.08	3	1
Total		19.17 ± 7.43	9	11

Values are mean ± standard deviation. T1: First 6 aligners; T2: Subsequent 6 aligners

**Fig. 1:**
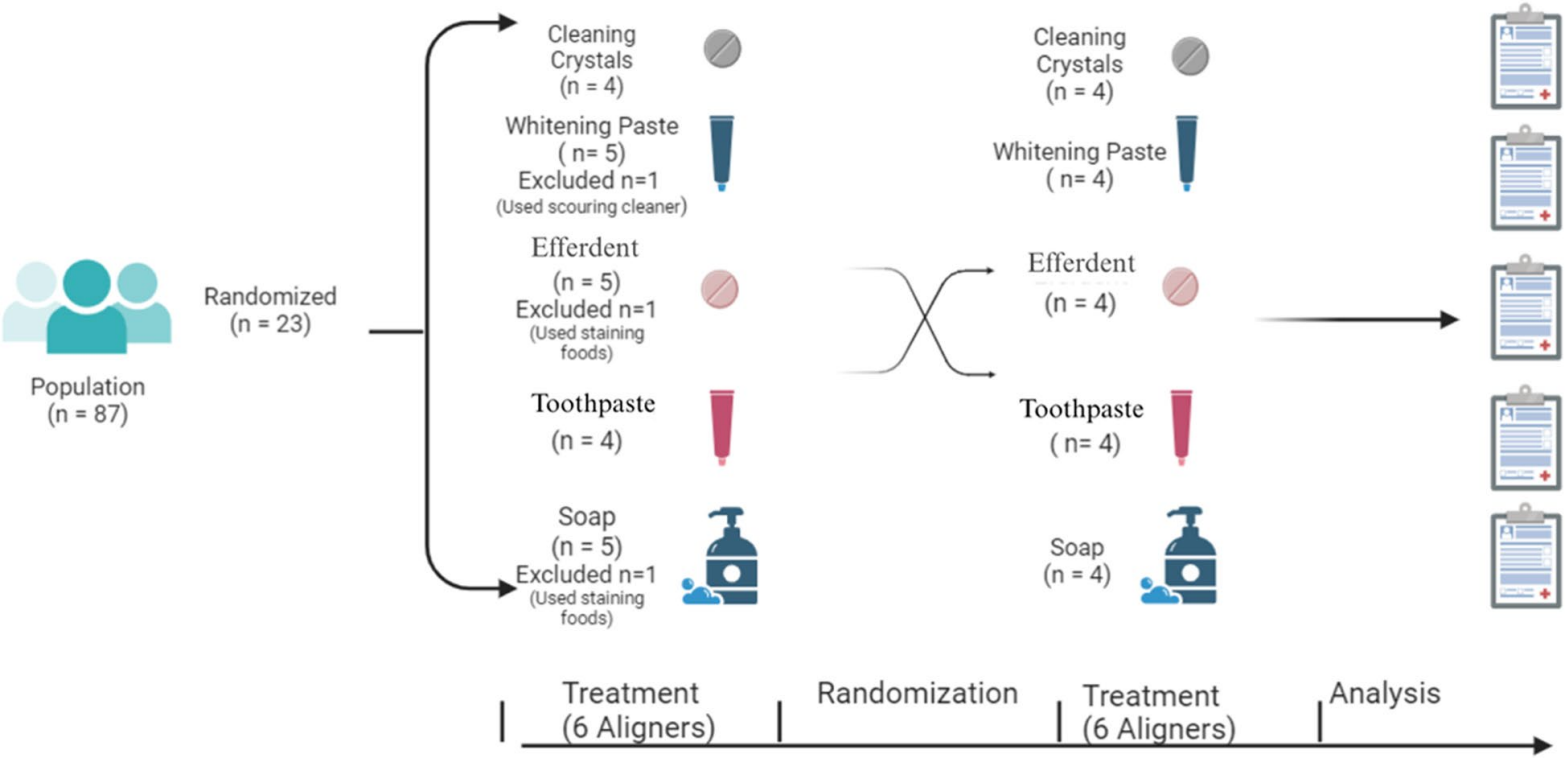
Visualization of study flow chart created with biorender.com

Participants were randomly assigned to five groups based on the cleaning agent used for aligner maintenance. Group 1 used Invisalign Cleaning Crystals, a product recommended by the aligner manufacturer. Group 2 used Efferdent Anti-Bacterial Denture Cleanser, a commonly available over-the-counter product. Group 3 used Sensodyne Rapid Relief toothpaste. Group 4 used Signal White Now Whitening Toothpaste, which contains active whitening agents. Group 5 used a colourant-free, transparent glycerin soap, which is both inexpensive and devoid of staining agents (Table 2, Fig. 1).

**Table 2:** Cleaning materials and usage methods

Used Material	Group Name	Product Usage
Invisalign™ Cleaning Crystals (Align Technology Inc, Tempe, USA)	Cleaning Crystals	Brushed with liquid soap (La Petit Marseillaise, Normandy, France) every time it was removed and before reinsertion. Soaked in Aligner Crystals dissolved in warm water for 15 min every 3 days
Signal White Now Whitening Toothpaste (Unilever, New Jersey, USA)	Whitening toothpaste	Brushed with Signal White Now toothpaste instead of liquid soap every time it was removed and before reinsertion
Efferdent Anti-Bacterial Denture Cleanser (Prestige Brands Inc, Lynchburg, USA)	Efferdent	Brushed with liquid soap(La Petit Marseillaise, Normandy, France) every time it was removed and before reinsertion. Soaked in Efferdent tablets dissolved in warm water for 15 min every 3 days
Sensodyne Rapid Relief toothpaste (GlaxoSmithKline, Brentford, UK)	Toothpaste	Brushed with Sensodyne toothpaste instead of liquid soap every time it was removed and before reinsertion
Colourant-free Clear Soap (La Petit Marseillaise, Normandy, France)	Liquid soap	Brushed with liquid soap every time it was removed and before reinsertion
Unused clear aligners	Control	After being taken out of aligner bags, clear aligners were rinsed with distilled water and dried using an air jet

### Randomization, assignment, and crossover design

**Fig. 2:**
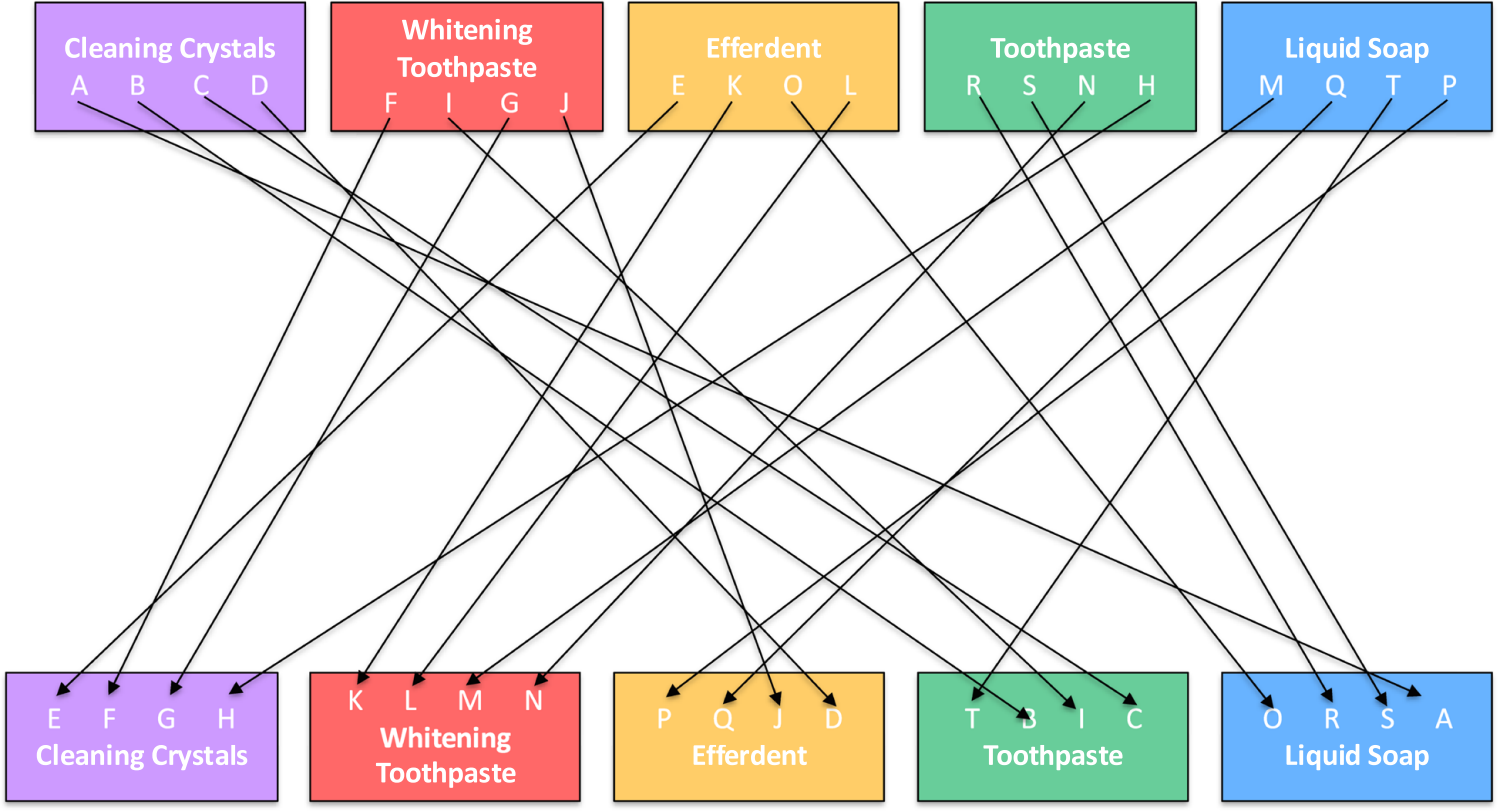
Crossover design of the study

Between October 2021 and June 2022, 20 patients who met the eligibility criteria and required more than 12 clear aligners were enrolled in the study. A crossover design was employed to enable within-subject comparisons (Fig. 2). Each participant was randomly assigned two different cleaning agents. For the first six aligners, participants were randomly allocated to one of five cleaning methods via lot drawing. Beginning with the seventh aligner, they switched to a second cleaning method, again determined by lot. In this second randomization, the initially assigned method and any group that had already reached its target sample size were excluded. This strategy ensured random assignment while maintaining balanced group distribution. Due to the requirement for uninterrupted orthodontic tooth movement, a washout period between the sixth and seventh aligners was not feasible.

Although each participant was assigned to use only two out of the five cleaning agents in a two-period crossover design, group allocation was controlled to ensure balanced sample sizes across treatment groups. Specifically, each cleaning agent was tested by 8 participants in total, with 4 participants applying it in the first period (T1) and 4 in the second period (T2). This approach maintained balance in the number of observations per treatment. However, the sequences of treatment administration (i.e., the order in which the two cleaning agents were used) were not fully balanced across participants, which may have introduced some variability related to period or sequence effects.

### Blinding

To maintain objectivity and minimize bias, blinding procedures were carefully implemented throughout the study. All randomization procedures, distribution of cleaning agents, provision of cleaning instructions, and collection of used aligners were carried out by the same orthodontist. However, all laboratory analyses related to colour change and surface roughness were performed by a different investigator who was blinded to both the patient identities and group assignments. This ensured that the evaluations were conducted in an unbiased and independent manner.

Participants were informed that the study aimed to investigate the effects of different cleaning methods on clear aligners; however, no specific information regarding the outcomes being assessed was provided. The informed consent form signed by the participants stated only: “A study will be conducted to examine changes occurring in treatment-purpose aligners depending on the cleaning methods used. For this purpose, patients will be provided with different cleaning agents and asked to clean their aligners accordingly.”

### Brushing and cleaning protocol

To minimize variability in surface roughness and to ensure the consistency of brushing practices across all participants, a standardized protocol was implemented. Each participant was provided with a commercially available, medium-soft toothbrush of the same brand and model. Participants were instructed to brush their clear aligners gently for approximately 60 s using light pressure, at least twice daily and after every meal. This protocol ensured uniform mechanical action on the aligner surfaces, reducing the influence of individual brushing habits. By maintaining consistency in the type of toothbrush and brushing technique, the observed differences in surface roughness and discoloration were attributable primarily to the cleaning agents used, rather than variations in mechanical cleaning practices.

### Cleaning agents and pH values

To further characterize the chemical environment to which the aligners were exposed, the pH levels of all cleaning agents used in this study were measured. The following pH values were recorded: Invisalign™ Cleaning Crystals – pH: 10.55; Efferdent Anti-Bacterial Denture Cleanser – pH: 5.41; Colourant-Free Clear Soap – pH: 6.41; Sensodyne Rapid Relief Toothpaste – pH: 6.66; and Signal White Now Whitening Toothpaste – pH: 6.88. These values have been included to provide a more comprehensive understanding of the potential chemical interactions influencing the surface characteristics of the aligners.

### Colour measurement

**Fig. 3:**
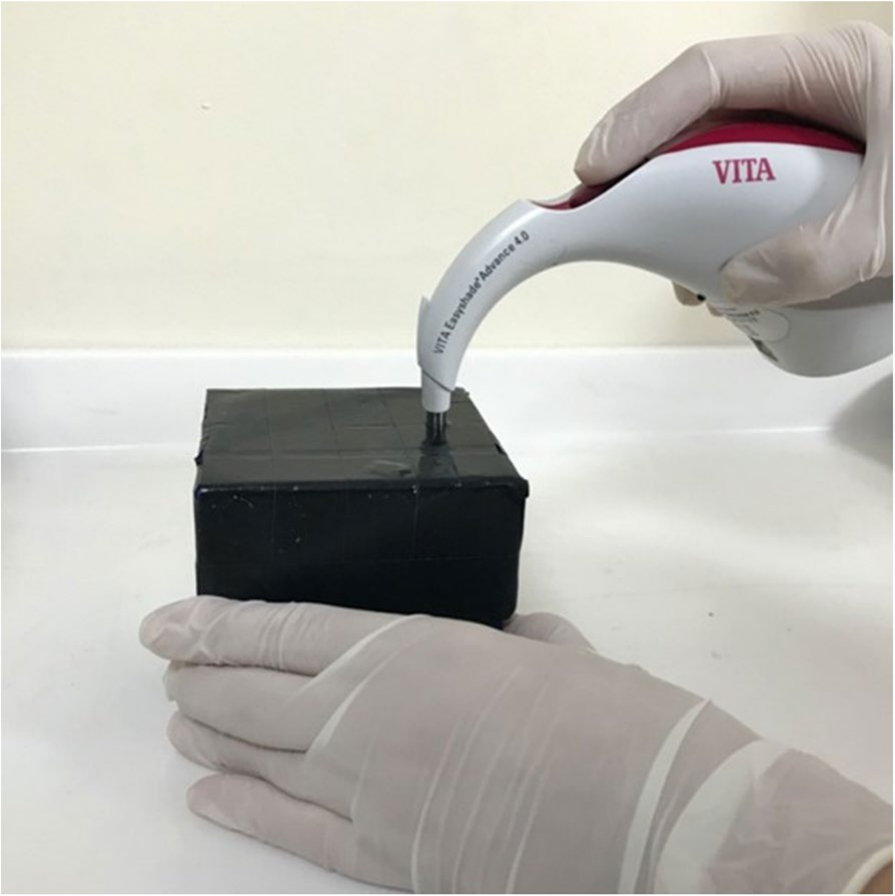
System for standard lighting and colour measurement from a single point

The colour measurement was carried out using the VITA Easyshade Compact spectrophotometer. To prevent external light interference and ensure consistent positioning, a custom-made cubic box with a 3 mm diameter entrance was used. The spectrophotometer head was secured to the lid of the box with black tape to enhance light-tightness (Fig. 3). Measurements were performed from the occlusal surface of the second premolar region on the aligners, which were placed against a standard white background. This region was selected to avoid areas with printed text or mandibular advancement (MA) extensions, which could interfere with accurate colour assessment, and to ensure reproducibility. Although anterior regions might show localized discoloration due to factors like oral breathing or gingival bleeding, the second premolar region was deemed the most clinically representative and reliable site for standardized evaluation.

The use of the Vita Easyshade spectrophotometer for assessing colour in transparent aligners is supported by relevant literature. Venkatasubramanian et al. demonstrated the effectiveness of this device in detecting colour changes in aligner materials exposed to various staining agents in an in-vitro study [15]. Additionally, Knezović et al. validated the accuracy and repeatability of the VITA Easyshade® Advance 4.0 in both in vivo and in vitro conditions, confirming its reliability in dental shade matching [16].

Prior to each measurement, the device was calibrated according to the manufacturer's instructions. Each sample was measured three times, and the average was recorded. The Commission Internationale de l'Eclairage (CIE) Lab* system was employed for colour measurement. Colour differences were calculated using the ΔE formula (Formula 1), derived from the differences in L*, a*, and b* values between baseline and post-treatment measurements:

Formula.

1 $$\triangle\mathrm E=\left[\left({\mathrm L}_1-{\mathrm L}_0\right)^{{}^2}\;+\;\left({\mathrm a}_1-{\mathrm a}_0\right)^2\;+\;\left({\mathrm b}_1-{\mathrm b}_0^2\right)\right]^{1/2}$$

To assess the clinical relevance of colour change, the ΔE values were converted to National Bureau of Standards (NBS) units using the following equation [17–19]: NBS units = ΔE × 0.92.

### Surface roughness analysis

Surface roughness was assessed using an optical profilometer (MarSurf M 300 C; Mahr GmbH, Göttingen, Germany) equipped with a 5 µm radius diamond stylus. Labial segments from the central incisor region of each aligner were sectioned to obtain standardized samples. To ensure a uniform measuring surface, all specimens were mounted flat. Each sample was scanned in three different directions at three distinct points to reduce orientation bias. Measurements were performed at a stylus speed of 0.5 mm/s, with a traversing length (Lt) of 1.75 mm and a cut-off length of 0.25 mm, following EN ISO 4288 guidelines [20]. The average surface roughness (Ra, µm) was calculated from the repeated measurements, and values were documented for statistical analysis. The surface roughness of unused aligners served as the control condition for roughness comparisons.

### Light microscopic surface evaluation

After the intraoral use of each aligner, surface morphology was evaluated using light microscopy to assess structural integrity and surface contamination. A stereomicroscope (Eclipse Ni, Nikon Corporation, Tokyo, Japan) was used at × 4 magnification. All aligners were rinsed with distilled water and air-dried prior to imaging. Representative areas from the buccal surface of each aligner were photographed under standardized lighting conditions. Images were analyzed qualitatively to identify surface features such as scratches, grooves, biofilm residues, and debris accumulation. No staining or surface treatment was applied to the specimens before observation.

### Statistical analysis

First, we examined the differences in colour and surface roughness between groups using linear mixed models (LMM).

Next, the interaction effects of Group, Period (T1 vs. T2), and Arch (upper vs. lower) were analyzed using LMMs with Patient ID specified as a random effect. In the model, Group, Period, and Sequence were included as fixed effects to account for the crossover design.

Then, because we found significant main effects for Group, and significant interaction effects for Group × Period and Group × Arch, we performed post hoc pairwise comparisons with Bonferroni correction.

Patient ID was included as a random effect to account for repeated measures. Fixed effects included cleaning agent group (Efferdent, Cleaning Crystals, Whitening Toothpaste, Toothpaste, Liquid Soap), period (T1: first 6 aligners; T2: next 6 aligners), and arch (upper, lower), along with their two-way and three-way interactions. Type III tests were used to evaluate omnibus effects. Bonferroni-corrected post hoc pairwise comparisons were conducted where significant effects were detected. All analyses were conducted using Jamovi version 2.6.26, and statistical significance was set at p < 0.05.

Model assumptions and validation statistics, including R^2^ values, intraclass correlation coefficient (ICC), and residual normality testing, are provided in Additional file 1.

## Results

**Fig. 4:**
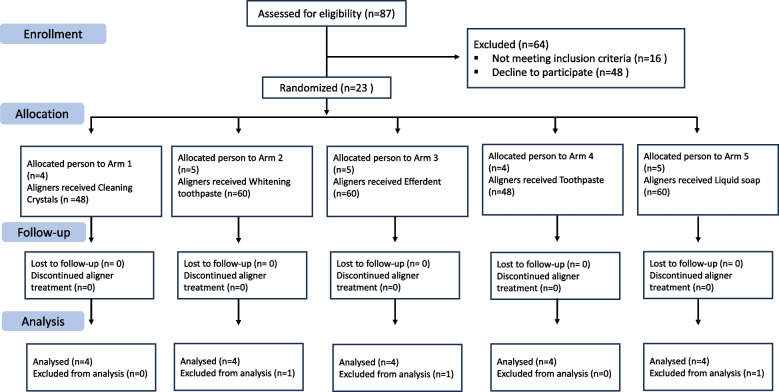
Flow diagram of the study design and participant allocation

A total of 23 patients were initially assessed for eligibility during the study period. However, the study commenced with 20 patients who each had more than 12 planned aligners (Fig. 4).

Two participants were excluded from the study: one due to frequent consumption of turmeric and curry, which caused staining unrelated to the assigned cleaning agents, and another for using an unapproved cleaning product (Cif Cream Cleaner) despite instructions.

Another patient was excluded due to both excessive coffee consumption and failure to adhere to the prescribed cleaning protocol (*n* = 1). All exclusions were made in accordance with predefined criteria. To maintain group balance, suitable replacement participants who met all eligibility criteria and provided informed consent were recruited and randomly assigned to the corresponding groups. As a result, the final analysis included 20 participants, evenly distributed across the study groups.

### Colour change (ΔE)

They were compared with aligners before usage and examined for changes in colour and surface roughness. The upper and lower aligners were also compared within each group to identify the region that was more prone to discolouration and deformation (Table 3).

**Table 3:** Pairwise comparisons of colour change between aligner cleaning agents

**Comparison**							
**Group Name**	**vs**	**Group Name**	**Difference**	**SE**	**t**	**df**	**p**_**bonferroni**_
**Efferdent**	**-**	**Cleaning Crystals**	**−12.873**	**2.07**	**−6.227**	**198**	** <.001**
**Efferdent**	**-**	**Toothpaste**	**−13.742**	**2.17**	**−6.319**	**192**	** <.001**
Efferdent	-	Liquid Soap	−3.976	1.95	−2.04	217	0.426
**Efferdent**	**-**	**Whitening Toothpaste**	**−8.002**	**1.94**	**−4.13**	**218**	** <.001**
Cleaning Crystals	-	Toothpaste	−0.869	1.94	−0.449	218	1
**Cleaning Crystals**	**-**	**Liquid Soap**	**8.897**	**2.12**	**4.195**	**194**	** <.001**
Cleaning Crystals	-	Whitening Toothpaste	4.871	2.03	2.396	206	0.175
**Toothpaste**	**-**	**Liquid Soap**	**9.766**	**1.95**	**5.01**	**217**	** <.001**
Toothpaste	-	Whitening Toothpaste	5.74	2.07	2.777	198	0.06
Liquid Soap	-	Whitening Toothpaste	−4.026	2.12	−1.898	194	0.592

Pairwise comparisons of mean NBS values between different cleaning agents are presented. Negative differences indicate that the first group listed had lower mean NBS values (i.e., less discoloration). Bonferroni-adjusted *p*-values are reported to account for multiple testing

*Abbreviations*: *SE* standard error, *df* degrees of freedom

Statistically significant differences are highlighted by p < 0.05 *Significant differences (p < 0.05, Bonferroni-adjusted) are shown in bold.

**Fig. 5:**
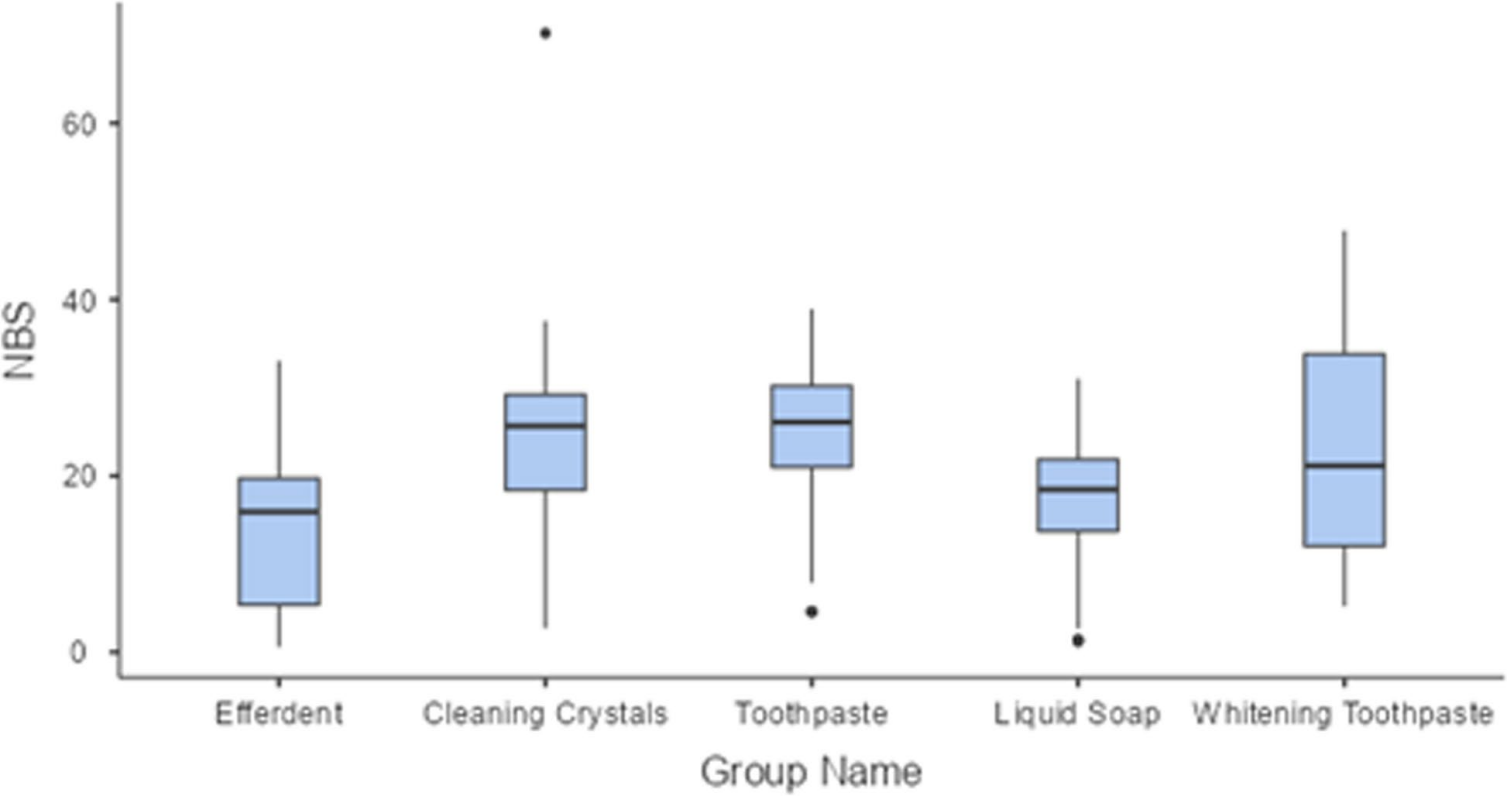
Box plot of colour change measurements among groups

A significant main effect of cleaning agent type on colour change was observed (F = 13.92, *p* < 0.001), indicating that the type of cleaning material used significantly affected discoloration levels across aligners. Aligners cleaned with Efferdent exhibited the lowest NBS scores, showing significantly less discoloration compared to Cleaning Crystals (*p* < 0.001), Toothpaste (*p* < 0.001), and Whitening Toothpaste (*p* < 0.001) (Fig. 5).

The main effects of period (T1 vs. T2) and arch (upper vs. lower) were not statistically significant (*p* = 0.502 and *p* = 0.335, respectively), suggesting that overall discoloration did not differ significantly across treatment periods or arch location in isolation.

**Fig. 6:**
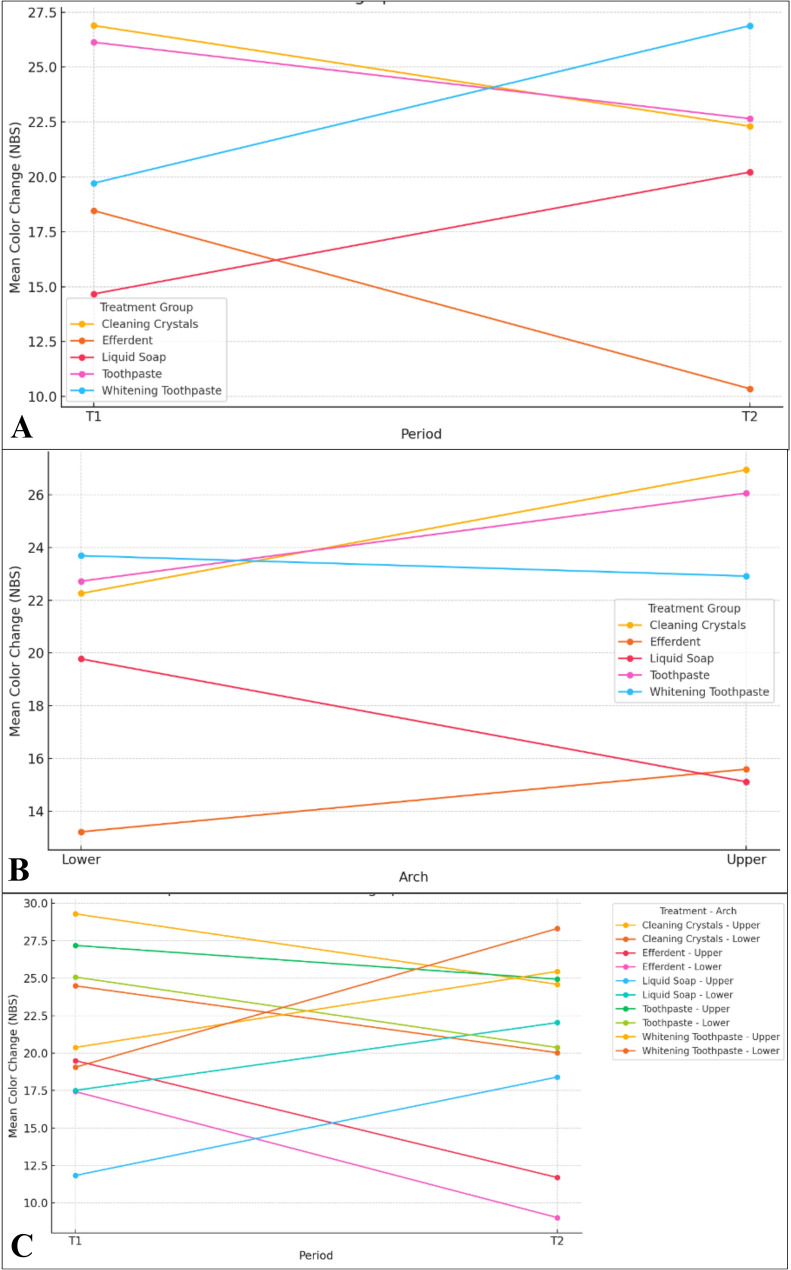
Line plots showing mean colour change by period and arch. (A) Mean colour change values for each treatment group across the two study periods (T1 and T2). (B) Mean colour change values strfatified by arch type (upper vs. lower) for each cleaning agent. (c) Combined visualization of colour change stratified by both period and arch, providing a comprehensive overview of treatment x period x arch interactions

However, a significant interaction was found between cleaning agent and period (*p* < 0.001), indicating that the impact of cleaning materials on discoloration varied depending on whether they were used in T1 or T2 (Table 4). Descriptive statistics revealed variation in mean discoloration across periods and arches for each treatment. Summary data for each treatment across periods (T1 and T2) are presented in Supplementary Table 1, Additional file 2, while arch-specific (upper vs. lower) mean discoloration values are reported in Supplementary Table 2, Additional file 3. Whitening Toothpaste and Liquid Soap showed higher values at T2 compared to T1, while Efferdent showed a decrease. Colour change also differed between upper and lower aligners within treatment groups, indicating a possible treatment-by-period interaction. These trends are summarized in the line chart in Fig. 6.

**Table 4:** Interaction between Cleaning Agent and Period on Colour Change

Comparison									
**Group Name**	**Period**	**vs**	**Group Name**	**Period**	**Difference**	**SE**	**t**	**df**	**p**_**bonferroni**_
Efferdent	T1	-	Cleaning Crystals	T1	−14.2575	3.31	−4.3072	126	0.001
Efferdent	T1	-	Toothpaste	T1	−15.9185	3.07	−5.192	196	<.001
Efferdent	T1	-	Whitening Toothpaste	T2	−9.4154	2.64	−3.5662	220	0.02
Efferdent	T2	-	Cleaning Crystals	T1	−19.3549	2.95	−6.5538	195	<.001
Efferdent	T2	-	Cleaning Crystals	T2	−11.4882	3.25	−3.5378	143	0.025
Efferdent	T2	-	Toothpaste	T1	−21.0158	3.34	−6.2879	119	<.001
Efferdent	T2	-	Toothpaste	T2	−11.5662	3.07	−3.7725	196	0.01
Efferdent	T2	-	Liquid Soap	T2	−12.043	3.31	−3.6382	126	0.018
Efferdent	T2	-	Whitening Toothpaste	T2	−14.5128	3.26	−4.451	136	<.001
Cleaning Crystals	T1	-	Liquid Soap	T1	18.3479	3.12	5.8834	183	<.001
Cleaning Crystals	T1	-	Whitening Toothpaste	T1	12.7661	3.17	4.0216	167	0.004
Toothpaste	T1	-	Liquid Soap	T1	20.0089	3.31	6.0447	126	<.001
Toothpaste	T1	-	Liquid Soap	T2	8.9729	2.64	3.3986	220	0.036
Toothpaste	T1	-	Whitening Toothpaste	T1	14.427	3.25	4.4428	143	<.001
Toothpaste	T2	-	Liquid Soap	T1	10.5592	2.95	3.5755	195	0.02
Liquid Soap	T1	-	Liquid Soap	T2	−11.036	3.31	−3.3326	131	0.05
Liquid Soap	T1	-	Whitening Toothpaste	T2	−13.5058	3.03	−4.4632	161	<.001

This table reports pairwise comparisons from a linear mixed model examining the interaction between cleaning agent groups and treatment period (T1: first six aligners; T2: subsequent six aligners) on colour change, measured as NBS values. Comparisons are presented as mean differences, standard errors (SE), t-values, degrees of freedom (df), and Bonferroni-adjusted *p*-values Only statistically significant comparisons (p < 0.05) are shown; non-significant comparisons were omitted

To further explore this interaction and mitigate potential carryover effects, we conducted a sensitivity analysis using only the T1 data. The T1-only model confirmed significant differences between cleaning agents (*p* < 0.001), with Cleaning Crystals and Toothpaste inducing more discoloration than Liquid Soap. Detailed pairwise comparisons are presented in Table 5.

Additionally, the interaction between cleaning agent and arch was also significant (*p* = 0.034), indicating that some cleaning methods demonstrated differential efficacy on upper or lower aligners (Table 5). Whitening Toothpaste caused significantly more discoloration in the upper arch compared to Efferdent and Liquid Soap, particularly during T2 (Table 5).

**Table 5:** Post hoc comparisons for cleaning agent × arch interaction on colour change

Comparison									
**Group Name**	**Arch**	**vs**	**Group Name**	**Arch**	**Difference**	**SE**	**t**	**df**	**p**_**bonferroni**_
Efferdent	Upper	-	Cleaning Crystals	Upper	−14.029	2.63	−5.3353	219	<.001
Efferdent	Upper	-	Cleaning Crystals	Lower	−9.345	2.63	−3.5539	219	0.021
Efferdent	Upper	-	Toothpaste	Upper	−14.225	2.71	−5.2398	217	<.001
Efferdent	Upper	-	Toothpaste	Lower	−10.888	2.71	−4.0106	217	0.004
Efferdent	Lower	-	Cleaning Crystals	Upper	−16.401	2.63	−6.2373	219	<.001
Efferdent	Lower	-	Cleaning Crystals	Lower	−11.717	2.63	−4.4559	219	<.001
Efferdent	Lower	-	Toothpaste	Upper	−16.597	2.71	−6.1134	217	<.001
Efferdent	Lower	-	Toothpaste	Lower	−13.26	2.71	−4.8842	217	<.001
Efferdent	Lower	-	Whitening Toothpaste	Upper	−8.802	2.53	−3.4805	218	0.027
Efferdent	Lower	-	Whitening Toothpaste	Lower	−9.574	2.53	−3.786	218	0.009
Cleaning Crystals	Upper	-	Liquid Soap	Upper	13.567	2.67	5.0775	218	<.001
Cleaning Crystals	Upper	-	Liquid Soap	Lower	8.911	2.67	3.335	218	0.045
Cleaning Crystals	Lower	-	Liquid Soap	Upper	8.882	2.67	3.3244	218	0.047
Toothpaste	Upper	-	Liquid Soap	Upper	13.763	2.54	5.4226	219	<.001
Toothpaste	Upper	-	Liquid Soap	Lower	9.107	2.54	3.5882	219	0.018
Toothpaste	Lower	-	Liquid Soap	Upper	10.425	2.54	4.1078	219	0.003

Pairwise comparisons from a linear mixed model evaluating how colour change (NBS units) differs between dental arches (upper vs. lower) for each cleaning agent. The table reports mean differences, standard errors (SE), t-values, degrees of freedom (df), and Bonferroni-adjusted *p*-values

Only statistically significant comparisons (*p* < 0.05) are shown; non-significant comparisons were omitted

The three-way interaction (group × period × arch) was not statistically significant (*p* = 0.258), indicating that the combined influence of all three factors did not produce additional variation beyond their pairwise effects (see Supplementary Table 3, Additional file 4).

### Surface roughness (Ra)

All assumptions for the linear mixed model were met, and model validation confirmed the appropriateness of the statistical approach.

Omnibus tests show that cleaning agent group (F = 7.07, *p* < 0.001) and period (T1 vs. T2; F = 9.73, *p* = 0.002) had significant main effects on surface roughness. Region (upper vs. lower arch) did not show a significant main effect but interacted significantly with period (F = 2.65, *p* = 0.014), indicating region-dependent effects over treatment phases.

**Fig. 7:**
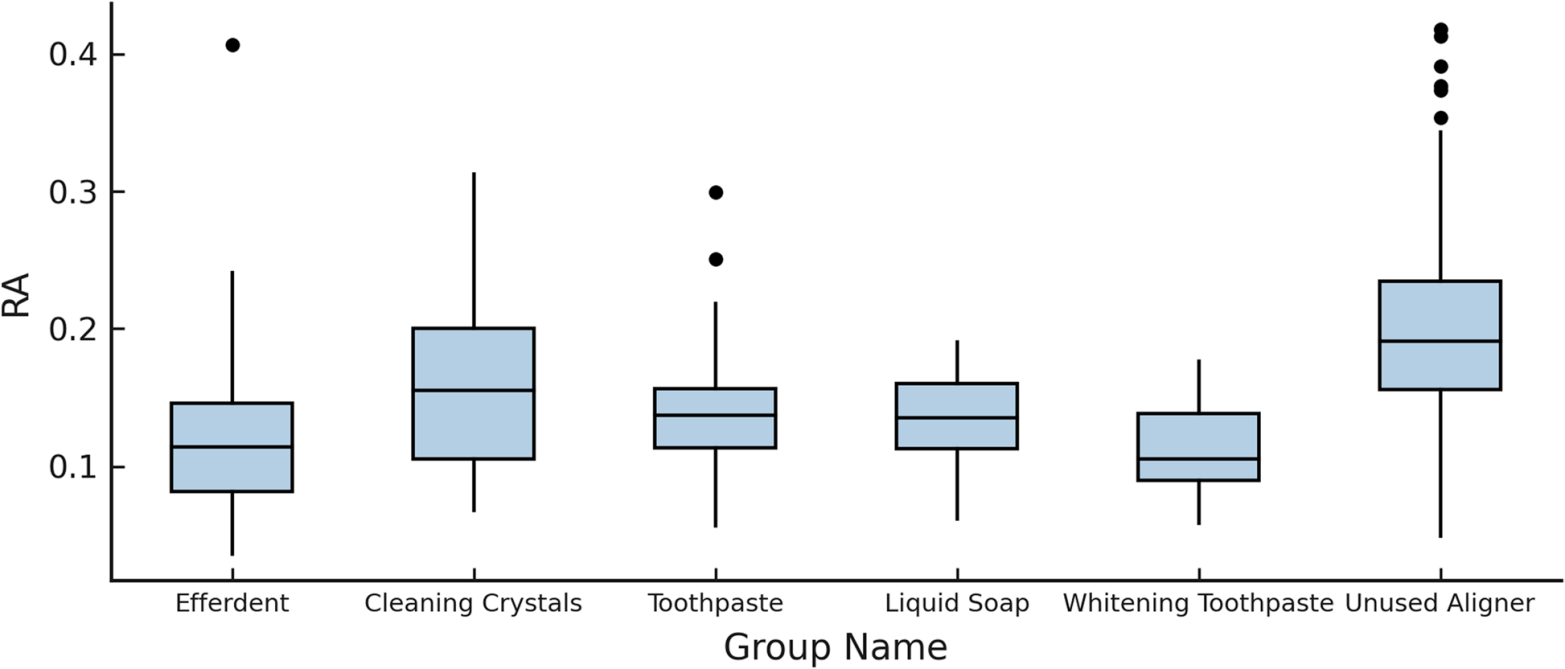
Box plot of surface roughness (Ra) values among groups

Table 6 summarizes pairwise post hoc comparisons of surface roughness (Ra) across cleaning agents. Whitening Toothpaste significantly reduced surface roughness compared to Toothpaste (mean difference = –0.03415 µm, *p* = 0.026), suggesting a more pronounced abrasive or smoothing effect on aligner surfaces. Whitening Toothpaste also resulted in lower Ra values compared to Efferdent (mean difference = –0.03784 µm, *p* = 0.004). Similarly, Cleaning Crystals showed significantly lower surface roughness than Efferdent (mean difference = –0.03368 µm, *p* = 0.011). No other comparisons reached statistical significance (Fig. 7).

**Table 6:** Pairwise comparisons of surface roughness between cleaning agents

**Comparison**							
**Group Name**	**vs**	**Group Name**	**Difference**	**SE**	**t**	**df**	**p**_**bonferroni**_
Efferdent	-	Cleaning Crystals	−0.04907	0.0111	−4.4314	140	<.001
Efferdent	-	Toothpaste	−0.03321	0.0116	−2.8571	120	0.050
Cleaning Crystals	-	Whitening Toothpaste	0.05001	0.0110	4.5473	152	<.001
Toothpaste	-	Whitening Toothpaste	0.03415	0.0111	3.0659	141	0.026

This table presents statistically significant pairwise comparisons from a linear mixed model evaluating the effect of different cleaning agents on surface roughness (Ra). Reported values include mean difference, standard error (SE), t-value, degrees of freedom (df), and Bonferroni-adjusted *p*-value. Negative values indicate lower roughness for the first group compared to the second. Only statistically significant comparisons (*p* < 0.05) are shown; non-significant comparisons were omitted

Surface roughness was significantly lower in T1 compared to T2 (difference = − 0.0185 µm, *p* = 0.002). There was no statistically significant difference in surface roughness between upper and lower arches (difference = − 0.00677 µm, *p* = 0.255). Significant differences in surface roughness were found only when Efferdent was used during T1, compared with several other treatment conditions (Table 7).

**Table 7:** Significant Pairwise Comparisons: Cleaning Agent × Treatment Period

**Comparison**									
**Group Name**	**Period**	**vs**	**Group Name**	**Period**	**Difference**	**SE**	**t**	**df**	**p**_**bonferroni**_
Efferdent	T1	-	Cleaning Crystals	T1	−0.07988	0.0170	−4.701	76.7	<.001
Efferdent	T1	-	Cleaning Crystals	T2	−0.07192	0.0156	−4.616	125.2	<.001
Efferdent	T1	-	Toothpaste	T2	−0.07018	0.0169	−4.150	73.4	0.004
Efferdent	T1	-	Liquid Soap	T2	−0.06552	0.0161	−4.081	124.0	0.004

This table presents only statistically significant (*p* < 0.05, Bonferroni-adjusted) pairwise comparisons between cleaning agents and treatment periods (T1: first six aligners; T2: subsequent six aligners). Ra refers to surface roughness in micrometres. Lower Ra values indicate greater surface smoothing. Non-significant comparisons have been excluded for clarity. Mean difference, standard error (SE), t-statistic, degrees of freedom (df), and adjusted *p*-values are shown

Significant differences in surface roughness were observed between specific cleaning agents and arch locations, with Efferdent (upper arch) producing significantly smoother surfaces than Cleaning Crystals, and Cleaning Crystals resulting in better-preserved surface morphology and lower surface roughness values than Whitening Toothpaste in both upper and lower arches (Table 8).

**Table 8:** Pairwise comparisons of cleaning agent × arch interaction on surface roughness

**Comparison**									
**Group Name**	**Arch**	**vs**	**Group Name**	**Arch**	**Difference**	**SE**	**t**	**df**	**p**_**bonferroni**_
Efferdent	Upper	-	Cleaning Crystals	Upper	−0.05547	0.0143	−3.8912	192	0.006
Efferdent	Upper	-	Cleaning Crystals	Lower	−0.05614	0.0143	−3.9389	192	0.005
Cleaning Crystals	Upper	-	Whitening Toothpaste	Upper	0.04902	0.0142	3.4529	197	0.031
Cleaning Crystals	Upper	-	Whitening Toothpaste	Lower	0.05033	0.0147	3.4339	198	0.033
Cleaning Crystals	Lower	-	Whitening Toothpaste	Upper	0.04969	0.0142	3.5011	197	0.026
Cleaning Crystals	Lower	-	Whitening Toothpaste	Lower	0.05100	0.0147	3.4806	198	0.028

This table presents statistically significant pairwise comparisons from a linear mixed model evaluating the interaction between cleaning agent type and dental arch (upper vs. lower) on surface roughness (Ra, µm). Each row reports the comparison of mean differences between specified groups, standard error (SE), t-values, degrees of freedom (df), and Bonferroni-adjusted *p*-values. Only comparisons with *p* < 0.05 are shown in this table

Significant pairwise comparisons between cleaning agent, treatment period, and arch on surface roughness are summarized in Supplementary Table 4, Additional file 5. Efferdent used during the first treatment phase (T1) on upper aligners resulted in significantly lower Ra values compared to Cleaning Crystals (T1 upper: *p* = 0.021; T1 lower: *p* = 0.002; T2 upper: *p* = 0.002; T2 lower: *p* = 0.029), Toothpaste (T2 upper: *p* = 0.018), and Liquid Soap (T2 upper: *p* = 0.007). A marginally significant difference was also observed when compared with Toothpaste T2 lower (*p* = 0.052).

### Light microscopic surface evaluation

**Fig. 8:**
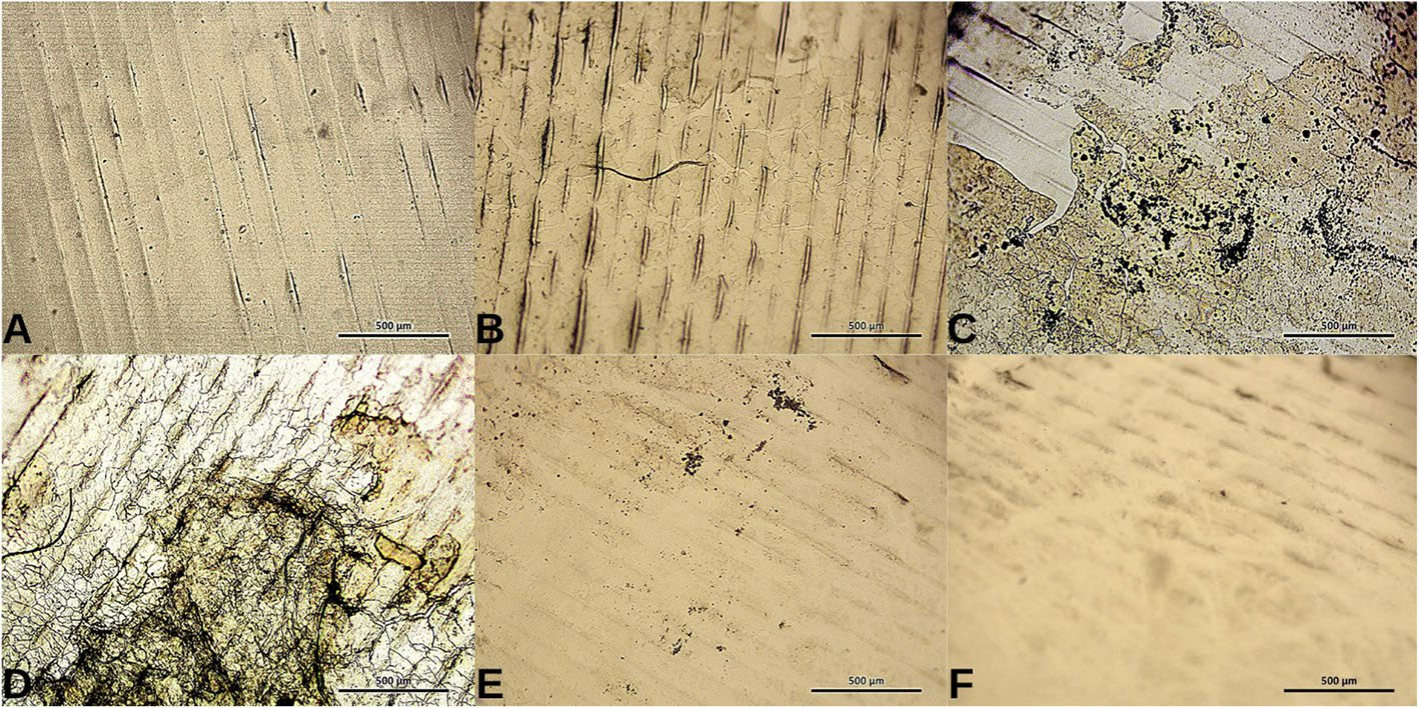
Light microscopic images of aligner surfaces after exposure to cleaning agents. Representative images are shown for each group: (**A**) Control, (**B**) Cleaning Crystals, (**C**) Whitening Toothpaste, (**D**) Efferdent, (**E**) Toothpaste, and (**F**) Liquid Soap. All images were captured at × 4 magnification; scale bar = 500 μm

Representative light microscopic images of the buccal surface of each aligner are shown in Fig. 8. The untreated control group (A) exhibited a uniform and continuous wavy surface pattern, with parallel ridges typical of the original manufacturing texture. No staining, scratches, or structural deterioration was observed.

The Cleaning Crystals group (B) demonstrated a diffuse surface discoloration covering a large area of the aligner. Unlike the discrete pigment accumulations seen in other groups, this group presented a more uniform, layer-like opacity. The original texture remained partially visible beneath this superficial layer, and no extensive mechanical surface breakdown was noted.

In the Whitening Toothpaste group (C), multiple regions exhibited clear signs of mechanical abrasion and surface cracks. These areas were interspersed with regions that retained their original texture. Pigment accumulation was evident, contributing to a patchy appearance.

The Efferdent group (D) showed extensive surface damage, with deep cracks and even fragmented areas where aligner material appeared partially lost. No pigment deposition was observed; however, the surface integrity was severely compromised throughout.

In the Toothpaste group (E), the original wavy surface pattern remained partially visible. However, numerous scattered pigment clusters were present, resulting in localized discoloration without major surface destruction.

The Liquid Soap group (F) displayed mostly preserved surface texture with mild, linear scratches. Surface discoloration was minimal and limited to faint areas. No cracking or loss of material was observed.

## Discussion

As an aesthetic treatment modality, orthodontic clear aligners must maintain their colour stability and transparency throughout the two-week wear period, as discoloration can result from plaque accumulation, bacterial biofilm, or pigment absorption into the material surface [1, 7, 21–24]. This study demonstrated that different cleaning agents significantly affected the colour and surface roughness of aligners after two weeks of intraoral use, leading to the rejection of the null hypothesis.

To minimize the impact of outliers on our analysis results, we have included individuals who did not overconsume food or drinks that contained colouring agents. Coffee and tea stains dental materials [18]. One of our participants had reported abstaining from coffee consumption during the enrollment phase. However, suspicions arose due to excessive deviation in ∆E measurement values, leading to our investigation revealing that the participant was consuming up to 3 cups of coffee and tea per day. This ultimately resulted in removing the patient from the liquid soap group. Also, another patient in the Efferdent group had excessive aligner discolouration due to regular consumption of curry containing food.

Due to the necessity of maintaining uninterrupted orthodontic tooth movement, a washout period between the sixth and seventh aligners could not be implemented. However, to minimize potential carryover effects, different cleaning agents were assigned to completely separate sets of aligners (first six vs. subsequent six). Each cleaning agent was used exclusively with a new set of aligners during its designated treatment period. Additionally, each cleaning material was used in both the early and late phases of treatment to balance potential sequence effects.

Despite these efforts, a statistically significant period effect was identified between T1 and T2, as reported in the results section. This may reflect a decline in patient compliance or changes in cleaning behavior during the second phase, possibly due to reduced motivation or habituation over time.

Another important consideration is the variation in baseline characteristics between periods, particularly age. As shown in Table 1, some treatment groups demonstrated notable age differences between T1 and T2. For example, in the liquid soap group, the average age of participants in T1 was 14.67 years, while in T2 it was 24.25 years. Such differences may have influenced compliance with instructions and oral hygiene habits, as younger participants may have required parental supervision or demonstrated lower adherence to the protocol. This imbalance may have introduced variability in outcomes and potentially confounded the main treatment effect.

Future studies should consider implementing age-stratified randomization or blocking, as well as a more balanced treatment sequence design (e.g., Latin square) to reduce period, sequence, and demographic-related confounding factors. Including a washout period, where clinically feasible, could also help to minimize carryover effects.

Crossover design allowed for a balanced and systematic distribution of each cleaning method across different periods of treatment, thereby aiming to minimize potential carry-over and period effects. We opted for a crossover design instead of a split-mouth design due to concerns about the accurate compliance of certain study participants, particularly adolescents, with the instructions of the split-mouth methodology. While split-mouth design has the distinct advantage of removing a significant amount of inter-subject variability from the estimated treatment effect necessitates, it requires a higher level of patient compliance [25, 26].

Colour difference formulas must provide a quantitative representation of the visual colour difference to be used in the practical application of technology that quantifies colour and colour differences in dentistry [27]. As an alternative to the NBS system, The CIEDE 2000 formula was developed in the year 2000 by international colour scientists, aiming to provide a more accurate and appropriate assessment of acceptability and perceptibility by considering a coefficient that influences the human visual perception more prominently. We opted for the NBS method due to its simplified calculation approach and its critical role in quality control and colour comparison tasks [28].

Whitening toothpaste resulted in a high level of colour change across both arches. It also produced the biggest surface alteration among the groups. High discoloration in this group is likely attributable to the presence of colourants (CI 74160 and CI 74260), which may adhere to the aligner surface. Light microscopy examinations confirmed degradation of the surface structure, with substantial surface-adherent material observed. The presence of abrasive components (such as hydrated silica and sodium lauryl sulfate) may account for both mechanical surface abrasion and enhanced pigment deposition, potentially leading to staining (Fig. 8C).

Efferdent, which had the most acidic pH among all cleaning agents, yielded the lowest NBS values, indicating minimal residual discoloration. However, it was also among the groups that caused the greatest reduction in surface roughness. This suggests that Efferdent may have chemically degraded the superficial layer of the aligner, effectively removing pigmented contaminants while simultaneously damaging the original material. Light microscopy supported this observation, revealing pronounced surface deterioration in Efferdent-treated aligners, with minimal residual pigment or foreign deposits (Fig. 8D).

Cleaning Crystals, formulated specifically for aligner care, caused high discoloration, but had a relatively limited impact on surface integrity, likely due to its milder chemical composition and alkaline pH.

Liquid soap resulted in the least overall change in both colour and surface texture, likely due to its mild, surfactant-based formulation free of abrasive or oxidising agents. However, its colour-cleaning efficacy declined from period T1 to period T2, suggesting that sustained effectiveness may depend on user compliance. The treatment-by-period interaction observed in our analysis suggests that the effects of liquid soap and whitening toothpaste vary across periods and should not be generalized. Reduced brushing diligence over time may have contributed to increased discoloration, as light microscopy revealed fine surface scratches likely due to cumulative brushing force (Fig. 8F). The greater discoloration caused by these agents in T2 may reflect cumulative effects or period-related influences. Despite replacing aligners every 10–14 days, residual pigment or material degradation from repeated exposure could accumulate, especially without a washout phase. Future studies should consider longer intervals or washout strategies to separate immediate from accumulated effects.

The significant interaction between treatment and period implies that treatment efficacy varied across time. Specifically, treatment effects were evident in T1 but diminished in T2, possibly due to carryover from the first intervention phase or period-related confounding factors such as decreased brushing compliance. Therefore, treatment-related conclusions should primarily be drawn from the first phase, and overall comparisons should be interpreted cautiously.

Although each cleaning agent was used across both treatment periods, we acknowledge that this does not eliminate the potential influence of treatment fatigue or ‘burn-out’ on patient compliance and cleaning behavior during the second phase. Such period-related effects may have biased the observed outcomes in T2 and confounded the true effect of the cleaning agents. Future studies with extended washout periods or alternative designs, such as Latin square or full crossover models with multiple sequences, are recommended to minimize these risks and better isolate the true agent-specific effects.

Although there are many studies examining the physical or chemical changes in Invisalign aligners in the literature, we did not come across any in vivo studies that evaluated the upper and lower aligners separately [6, 29]. In the context of aligner therapy, noticeable colour change can compromise the esthetic appeal of the appliance, one of the primary motivations for choosing clear aligners over fixed appliances. Our study found that all groups exhibited ΔE values greater than 12.0, which are considered “very marked” changes according to the NBS classification [17–19]. NBS values ranged from 14.4 (Efferdent) to 24.6 (Cleaning Crystals), which correspond to the highest category of visual detectability (“very much”) according to established scales [17–19]. Notably, values above 3.0 NBS units are generally regarded as clinically perceivable and potentially unacceptable, particularly in esthetically sensitive regions [17]. Therefore, even the best-performing group in terms of colour stability (Efferdent) exhibited changes that exceed clinically acceptable thresholds. Such levels of discoloration are likely to be visible to the human eye and may lead to patient dissatisfaction, reduced compliance, and possibly early replacement of aligners, which increases treatment cost and complexity. In light of these results, the liquid soap and efferdent groups demonstrated the lowest NBS scores, supporting their clinical viability as a low-impact cleaning method in terms of esthetics. However, the roughness values shown by the Efferdent group make this material not recommended.

Surface roughness, meanwhile, has direct implications for biofilm formation and hygiene [30]. Rougher surfaces facilitate plaque accumulation, increasing the risk of gingival inflammation, bad breath, and enamel demineralization during aligner wear [29]. Although all groups in our study remained below the 0.2 µm clinical threshold for critical roughness, some materials preserved the original surface texture more effectively. Notably, Cleaning Crystals and standard toothpaste maintained roughness values closer to the unused aligners, suggesting less harmful effect. Therefore, cleaning agents should not only be evaluated for their cleaning efficacy but also for their ability to preserve the integrity of aligners.

Patients may remove their aligners before consuming coloured substances, and then brush their teeth and aligners. However, it's important to note that residual staining agents can potentially remain on areas like the tongue, cheek mucosa, and palate, which could also contribute to changes in colour. Except for cleaning crystals, the cleaning agents tested in our study are not specifically designed for orthodontic aligners but are examples of alternative materials with the greatest potential for patient use. Despite providing patients with guidance and suggestions, orthodontists do not have complete control over the cleaning material preferences of the patients. This lack of control can pose challenges, as patient preferences can vary widely. So, clinicians should inform their patients about the potential consequences of using cleaning materials containing colourants and advise them to check the contents of the products they choose to use. Oral hygiene formulations consist of various ingredients such as, fluoride, detergents, flavoring agents, and thickening agents. Abrasives are also commonly used to remove debris and stains.

Iliadi et al. aimed to assess the performance of two types of clear aligners by testing them with three different cleaning agents [31]. Two of the agents were alkaline peroxide solutions called Retainer Brite and Retainer Cleaner, while the third agent, Steraligner, was peroxide-free. They observed oxidative effects in Retainer Brite, supporting that the polyester-urethane structure of Invisalign aligners might be more sensitive to sodium perborate. Although aligners cleaned with Efferdent tablets containing sodium perborate exhibited the least discoloration, this cleaning method was among those that caused the most substantial surface degradation. Even though our study results seem to be consistent with these findings, it is important to note that our study did not involve chemical analysis. Therefore, further research is warranted to investigate the potential effects of cleaning agents containing sodium perborate, such as Efferdent, on clear aligners.

The surface morphology of unused aligners from Invisalign company exhibits distinct characteristics when compared to other aligner brands, displaying a non-linear and non-uniform pattern consisting of regular and evenly distributed wrinkles [32]. For assessing the surface roughness of removable thermoplastic dental appliances, both Atomic Force Microscopy (AFM) and profilometry methods could be utilized. In comparison to the profilometer's 5 μm diamond stylus, the AFM has a 0.01 μm tip, allowing for more exact measurements [33]. The use of an optical profilometer, chosen for its high lateral resolution, speed, and reliability, allows for a wider range of amplitude measurements, calculation of various roughness parameters, and faster scanning compared to AFM, making it the preferred method for evaluating the 3D surface texture of removable thermoplastic dental appliances in our study [34, 35].

Based on the chosen methodology, our findings can be compared with previous research. Previous SEM investigations conducted at magnifications of 10–30 K reported that Invisalign exhibited the lowest degree of surface roughness among the materials examined [36]. However, in our light microscope observations, the surface of Invisalign polyurethane material displayed prominent undulations characterized by substantial irregularities (Fig. 8A). It is possible that the company intentionally employed manufacturing processes to create this wavy pattern, potentially aiming to enhance the flexibility of the material [29]. We observed that the unused aligners exhibited the highest level of surface roughness due to this original surface structure. Our finding is consistent with the findings of Papadopoulou et al., who demonstrated that the roughness variables of the as-received material were reduced after intraoral service, indicating a wear effect [37]. Therefore, groups with high roughness values had the least deformation and were closest to the original form. In groups where the roughness value decreased, there was visible wear on the surface under the light microscope. (Fig. 8C and 8D). These damages play a significant role in surface leveling, ultimately leading to a notable decrease in surface roughness, as supported by the findings of Papadopoulou et al. [35]. The Cleaning Crystals group, the brand's product, exhibited similar roughness values to the unused aligners for both upper and lower groups. Along with the toothpaste group, it effectively preserved the textured surface of the aligners. This finding suggests that the utilization of the recommended cleaning crystals by the company is an effective cleaning method that helps preserve the surface structure closest to its original rough and wavy state.

Light microscopic observations provided qualitative confirmation of the surface integrity results (Fig. 8). Whitening Toothpaste and Efferdent caused visible surface deterioration, with evidence of abrasion or erosion consistent with their chemical and abrasive compositions. Cleaning Crystals showed comparatively less damage, while Liquid Soap maintained near-intact surface topography with only minor brushing-related scratches. The intentional waviness observed in the unused aligners (Fig. 8A) further supports the hypothesis that the manufacturer employs surface engineering to enhance flexibility. These morphological findings align with the Ra values and provide a visual correlate to the quantitative surface roughness measurements.

A significant effect of arch type was observed in both colour change and surface roughness outcomes, indicating that upper and lower aligners responded differently to the tested cleaning agents. Several factors may contribute to this difference. Anatomically, the lower arch is more exposed to higher plaque accumulation and mechanical stress which may enhance staining and surface wear. It has been shown that the lower anterior teeth consistently accumulate significantly more plaque compared to the upper anterior teeth, regardless of tooth alignment, likely due to anatomical and salivary factors [38]. Furthermore, the mandibular anterior region tends to retain more saliva due to its proximity to major salivary gland openings, influencing salivary clearance and promoting higher plaque accumulation compared to the upper arch [39]. In contrast, the upper arch generally experiences less salivary influence, potentially resulting in less exposure to staining agents and mechanical stress.

Compliance differences could also play a role; patients may be more meticulous in cleaning the more visible upper aligners compared to lower ones. Moreover, minor differences in intraoral pH, temperature, and biofilm accumulation between arches may influence how materials degrade or interact with cleaning agents. The aligners’ fit and tightness, which can differ slightly between arches due to tooth anatomy and movement goals, may also affect the degree of exposure to external factors.

Although few studies have directly compared arch-specific responses to cleaning agents, Fang et al. (2020) reported variability in aligner material degradation and discoloration due to intraoral environmental factors, supporting the possibility that anatomical and functional differences between arches could influence treatment outcomes [40]. Future studies may benefit from analyzing arch-specific variables more comprehensively, potentially considering separate intervention strategies for upper and lower aligners.

### Strengths and limitations

Given the clinical nature of our study, we explored a limited set of materials, only five in number. The continuous introduction of new products in the market further limits our scope, allowing us to comment solely on the effects of the cleaning materials we have specifically examined. Thus, our study is constrained to providing insights exclusively into the impacts of the researched cleaning materials. Nevertheless, our work represents a pioneering effort, aiming to illuminate the considerations that dentists may consider when discussing or responding to questions about alternative cleaning agents. Although Group × Period interaction was statistically tested and found to be significant, the absence of a formal washout period limits the ability to fully isolate period effects inherent to the crossover design.

Although participants were given standardized instructions to store their aligners in the original Invisalign storage cases when not in use, we did not collect specific data on whether they consistently followed these guidelines. Improper storage, such as leaving aligners exposed to air or in non-recommended containers, could potentially contribute to changes in colour or surface characteristics due to environmental exposure (e.g., temperature, humidity, or contamination). Therefore, the lack of control over actual storage conditions may have introduced variability in the outcomes.

Another important limitation is the use of a two-period partial crossover design. While this approach allowed within-subject comparisons for two cleaning agents per participant, comparisons involving other cleaning agents were between-subject, which may have increased variability in the results. The balanced allocation of participants across treatment groups (*n* = 8 per group, 4 in each period) helped maintain comparability between groups; however, the treatment sequences were not evenly distributed among participants, potentially introducing confounding due to period or sequence effects. In addition, the lack of a washout period, which was not feasible due to the necessity of continuous orthodontic tooth movement may have contributed to carryover effects. Future research should consider employing a full crossover design with multiple periods and balanced treatment sequences, such as a Latin square or Williams design, so that each participant receives all tested treatments in randomized order. This approach would reduce variability related to period and sequence, minimize potential carryover effects, and increase the internal validity of the findings.

## Conclusion

This randomized crossover clinical trial demonstrated that different cleaning agents exert varying effects on both the colour stability and surface characteristics of Invisalign clear aligners. Clinically perceptible colour changes were observed in all groups, potentially compromising the esthetic quality of the aligners. Efferdent produced the least discoloration, followed closely by colorant-free liquid soap. However, because of its significant surface wear likely resulting from chemical deterioration, Efferdent is not advisable for frequent use, despite its ability to yield a visually clean appearance. Patients should be informed that such effects may compromise the aligner material.

While all tested agents resulted in surface roughness levels below the critical threshold for plaque accumulation, Cleaning Crystals and conventional toothpaste were most effective in preserving the original surface morphology. However, they did not sufficiently protect against discoloration.

Considering the constraints of the crossover study design, such as the absence of a washout period, possible carryover effects, and significant period and interaction effects, these findings should be interpreted with caution. The treatment effects observed in this study may not be fully generalizable and may vary depending on the specific period or contextual factors**.** Future long-term clinical trials using age-stratified randomization, washout phases, and more diverse participant samples are recommended to confirm and expand upon these findings, ultimately supporting the development of evidence-based guidelines for optimal aligner maintenance. 

## Supplementary Information

Additional file 1.

Additional file 2.

Additional file 3.

Additional file 4.

Additional file 5.

## Data Availability

We are committed to ensuring that our data collection, processing, and use are transparent and comply with applicable laws and regulations. We are committed to providing users with clear and unambiguous information. The data supporting the findings of this study are available from the corresponding author upon reasonable request. The data supporting the findings of this study are available from the corresponding author upon reasonable request.

## References

[1] Ziuchkovski JP, Fields HW, Johnston WM, et al. Assessment of perceived orthodontic appliance attractiveness. Am J Orthod Dentofacial Orthop. 2008;133:68–78. 10.1016/j.ajodo.2006.07.025. 10.1016/j.ajodo.2006.07.025 18407023

[2] Kuo E, Miller RJ. Automated custom-manufacturing technology in orthodontics. Am J Orthod Dentofacial Orthop. 2003;123:578–81. 10.1067/mod.2003.S0889540603000519. 12750680 10.1067/mod.2003.S0889540603000519

[3] Invisalign. Invisalign Cleaning System. San Jose (CA): Align Technology, Inc.; 2024. Available from: https://shop.invisalign.com/products/invisalign-cleaning-system.Cited 2025 May 3.

[4] Eichenauer J, Serbesis C, Ruf S. Cleaning removable orthodontic appliances—a survey. J Orofac Orthop. 2011;72:389–95. 10.1007/s00056-011-0043-2. 21990062 10.1007/s00056-011-0043-2

[5] Lombardo L, Martini M, Cervinara F, et al. Comparative SEM analysis of nine F22 aligner cleaning strategies. Prog Orthod. 2017;18:26. 10.1186/s40510-017-0178-9. 28782094 10.1186/s40510-017-0178-9 PMC5592163

[6] Schuster S, Eliades G, Zinelis S, et al. Structural conformation and leaching from in vitro aged and retrieved Invisalign appliances. Am J Orthod Dentofacial Orthop. 2004;126:725–8. 10.1016/j.ajodo.2004.04.021. 15592222 10.1016/j.ajodo.2004.04.021

[7] Gracco A, Mazzoli A, Favoni O, et al. Short-term chemical and physical changes in Invisalign appliances. Aust Orthod J. 2009;25:34–40. 19634462

[8] Shpack N, Greenstein RB, Gazit D, Sarig R, Vardimon AD. Efficacy of three hygienic protocols in reducing biofilm adherence to removable thermoplastic appliance. Angle Orthod. 2014;84:161–70. 23786595 10.2319/012413-75.1 PMC8683063

[9] Charavet C, Graveline L, Gourdain Z, Lupi L. What are the cleaning and disinfection methods for acrylic orthodontic removable appliance? A systematic review. Children. 2021;8(11):967. 34828679 10.3390/children8110967 PMC8623359

[10] Pardo A, Signoriello A, Zangani A, et al. Home biofilm management in orthodontic aligners: a systematic review. Dent J. 2024;12(10):335. 10.3390/dj12100335 PMC11506732 39452463

[11] Agarwal M, Wible E, Ramir T, et al. Long-term effects of seven cleaning methods on light transmittance, surface roughness, and flexural modulus of polyurethane retainer material. Angle Orthod. 2018;88:355–62. 10.2319/081517-551.1. 29509024 10.2319/081517-551.1 PMC8288323

[12] Bernard G, Rompré P, Tavares JR, et al. Colorimetric and spectrophotometric measurements of orthodontic thermoplastic aligners exposed to various staining sources and cleaning methods. Head Face Med. 2020;16:2. 10.1186/s13005-020-00218-2. 32070379 10.1186/s13005-020-00218-2 PMC7027305

[13] Alper Ö, Zeynep Ö, Canlı E, et al. Farklı estetik braketlerin renk stabilitelerinin karşılaştırılması. Ondokuz Mayıs Univ Diş Hekimliği Derg. 2012;13:7–12.

[14] Lira LF, Vargas EOA, Silva EM, et al. Effect of oral exposure on chemical, physical, mechanical, and morphologic properties of clear orthodontic aligners. Am J Orthod Dentofacial Orthop. 2023;164:e51-63. 10.1016/j.ajodo.2023.05.015. 37330727 10.1016/j.ajodo.2023.05.015

[15] Venkatasubramanian P, Jerome MS, Ragunanthanan L, et al. Color stability of aligner materials on exposure to indigenous food products: an in-vitro study. J Dent Res Dent Clin Dent Prospects. 2022;16(4):221. 37560499 10.34172/joddd.2022.035 PMC10407867

[16] Knezović D, Zlatarić D, Illeš IŽ, Alajbeg M. In vivo and in vitro evaluations of repeatability and accuracy of VITA Easyshade® Advance 4.0 dental shade-matching device. Acta Stomatol Croat. 2015;49(2):112–21. 10.15644/asc49/2/4. 10.15644/asc49/2/4 PMC4988825 27688393

[17] da Silva DL, Mattos CT, de Araújo MV, et al. Color stability and fluorescence of different orthodontic esthetic archwires. Angle Orthod. 2013;83:127–32. 10.2319/121311-764.1. 22591261 10.2319/121311-764.1 PMC8805526

[18] Filho HL, Maia LH, Araújo MV, et al. Colour stability of aesthetic brackets: ceramic and plastic. Aust Orthod J. 2013;29:13–20. 23785933

[19] Koksal T, Dikbas I. Color stability of different denture teeth materials against various staining agents. Dent Mater J. 2008;27:139–44. 10.4012/dmj.27.139. 18309623 10.4012/dmj.27.139

[20] International Organization for Standardization. Geometrical Product Specifications (GPS) – Surface texture: Profile method – Rules and procedures for the assessment of surface texture. ISO 4288:1996. Geneva: ISO; 1996. Available from: https://www.iso.org/standard/2096.html

[21] Low B, Lee W, Seneviratne C, et al. Ultrastructure and morphology of biofilms on thermoplastic orthodontic appliances in ‘fast’ and ‘slow’ plaque formers. Eur J Orthod. 2011;33:577–83. 10.1093/ejo/cjq126. 21187528 10.1093/ejo/cjq126

[22] Levrini L, Novara F, Margherini S, et al. Scanning electron microscopy analysis of the growth of dental plaque on the surfaces of removable orthodontic aligners after the use of different cleaning methods. Clin Cosmet Investig Dent. 2015;7:125–31. 10.2147/CCIDE.S95814. 26719726 10.2147/CCIDE.S95814 PMC4687621

[23] Liu CL, Sun WT, Liao W, et al. Colour stabilities of three types of orthodontic clear aligners exposed to staining agents. Int J Oral Sci. 2016;8:246–53. 10.1038/ijos.2016.25. 27660048 10.1038/ijos.2016.25 PMC5168413

[24] Inami T, Tanimoto Y, Minami N, et al. Color stability of laboratory glass-fiber-reinforced plastics for esthetic orthodontic wires. Korean J Orthod. 2015;45:130–5. 10.4041/kjod.2015.45.3.130. 26023541 10.4041/kjod.2015.45.3.130 PMC4446374

[25] Zhu H, Zhang S, Ahn C. Sample size considerations for split-mouth design. Stat Methods Med Res. 2017;26:2543–51. 10.1177/0962280215601137. 26303156 10.1177/0962280215601137 PMC5573650

[26] Pandis N, Walsh T, Polychronopoulou A, et al. Split-mouth designs in orthodontics: an overview with applications to orthodontic clinical trials. Eur J Orthod. 2013;35:783–9. 10.1093/ejo/cjs108. 23376899 10.1093/ejo/cjs108

[27] Perez MM, Ghinea R, Herrera LJ, et al. Dental ceramics: A CIEDE2000 acceptability thresholds for lightness, chroma and hue differences. J Dent. 2011;39(Suppl 3):e37-44. 10.1016/j.jdent.2011.09.007. 21986320 10.1016/j.jdent.2011.09.007

[28] Elraggal A, Alhotan A, Yates J, et al. Effect of different solutions on the colour stability of nano-particles or fibre reinforced PMMA. Polymers. 2022;14(8):1521. 10.3390/polym14081521. 35458269 10.3390/polym14081521 PMC9028232

[29] Fang D, Li F, Zhang Y, et al. Changes in mechanical properties, surface morphology, structure, and composition of Invisalign material in the oral environment. Am J Orthod Dentofacial Orthop. 2020;157:745–53. 10.1016/j.ajodo.2019.05.023. 32487304 10.1016/j.ajodo.2019.05.023

[30] Bollen CM, Lambrechts P, Quirynen M. Comparison of surface roughness of oral hard materials to the threshold surface roughness for bacterial plaque retention: a review of the literature. Dent Mater. 1997;13:258–69. 11696906 10.1016/s0109-5641(97)80038-3

[31] Iliadi A, Enzler V, Polychronis G, et al. Effect of cleansers on the composition and mechanical properties of orthodontic aligners in vitro. Prog Orthod. 2022;23:54. 10.1186/s40510-022-00449-w. 36517652 10.1186/s40510-022-00449-w PMC9751251

[32] Daniele V, Macera L, Taglieri G, et al. Color stability, chemico-physical and optical features of the most common PETG and PU based orthodontic aligners for clear aligner therapy. Polymers. 2021;14:14. 10.3390/polym14010014. 35012036 10.3390/polym14010014 PMC8747426

[33] Porojan L, Vasiliu RD, Porojan SD, et al. Surface quality evaluation of removable thermoplastic dental appliances related to staining beverages and cleaning agents. Polymers. 2020;12(8):1736. 10.3390/polym12081736. 32756439 10.3390/polym12081736 PMC7464035

[34] Mei L, Guan G. Profilometry and atomic force microscopy for surface characterization. Nano TransMed. 2023;2:e9130017. 10.26599/NTM.2023.9130017.

[35] Papadopoulou AK, Cantele A, Polychronis G, et al. Changes in roughness and mechanical properties of Invisalign® appliances after one- and two-week use. Materials. 2019;12:2406. 10.3390/ma12152406. 31357697 10.3390/ma12152406 PMC6696190

[36] Alhendi A, Khounganian R, Ali R, et al. Structural conformation comparison of different clear aligner systems: an in vitro study. Dent J. 2022;10:73. 10.3390/dj10050073. 10.3390/dj10050073 PMC9139589 35621526

[37] Morton J, Derakhshan M, Kaza S, et al. Design of the Invisalign system performance. Semin Orthod. 2017;23:3–11.

[38] Griffiths GS, Addy M. Effects of malalignment of teeth in the anterior segments on plaque accumulation and gingivitis. J Clin Periodontol. 1981;8(6):481–90. 10.1111/j.1600-051X.1981.tb02024.x. PMID: 6949921. 6949921 10.1111/j.1600-051x.1981.tb00897.x

[39] Alhajj M, Babos M. Physiology, Salivation. In: StatPearls. Treasure Island (FL): StatPearls Publishing; 2023. Available from: https://www.ncbi.nlm.nih.gov/books/NBK542251/31194408

[40] Fang Y, Zhao Z, Zheng W. Advances in orthodontic clear aligner materials: Mechanical properties and degradation behaviors under intraoral conditions. Materials. 2020;13(8):1896. 10.3390/ma13081896. 32316517 10.3390/ma13081896 PMC7216083

