# Supplementary material for: Comparative evaluation of the effect of different cleaning agents on colour and surface roughness of Invisalign clear aligners: a cross-over randomized controlled trial
Source: BMC Oral Health. 2025 Nov 4;25:1745. doi: 10.1186/s12903-025-06928-w (PMC12584337; doi:10.1186/s12903-025-06928-w)
Supplement: Supplementary file 1 — Additional file 1. [file 12903_2025_6928_MOESM1_ESM.docx]

Statistical Model Assumptions and Validation

The marginal R² value indicates that fixed effects explain 22.8% of the variance, while the conditional R² value shows that fixed and random effects together explain 34.6% of the variance. The likelihood ratio test (LRT) was significant (p < 0.001), indicating the importance of including fixed effects.

The intraclass correlation coefficient (ICC) was 0.153, suggesting that approximately 15% of the variance in surface roughness was due to differences between patients. This supports the use of a random intercept model. Random effect significantly improved model fit (AIC = -582; LRT = 8.51, p = 0.004).

Normality of residuals was evaluated using the Kolmogorov–Smirnov test. The test was not significant (p = 0.176), supporting the assumption of normality.
