# Supplementary material for: Comparative evaluation of the effect of different cleaning agents on colour and surface roughness of Invisalign clear aligners: a cross-over randomized controlled trial
Source: BMC Oral Health. 2025 Nov 4;25:1745. doi: 10.1186/s12903-025-06928-w (PMC12584337; doi:10.1186/s12903-025-06928-w)
Supplement: Supplementary file 2 — Additional file 2. [file 12903_2025_6928_MOESM2_ESM.docx]

Table S1 Descriptive table that report mean color change for each treatment in each period.

| MEAN NBS | | |
| --- | --- | --- |
| Group Name | **T1** | **T2** |
| Efferdent | 18.5 | 10.4 |
| Cleaning Crystals | 26.9 | 22.3 |
| Toothpaste | 26.1 | 22.7 |
| Liquid Soap | 14.7 | 20.2 |
| Whitening Toothpaste | 19.7 | 26.9 |

Note. This table presents the mean color change values, expressed in National Bureau of Standards (NBS) units, for each cleaning agent during the two study periods (T1 and T2). T1 and T2 refer to the first and second phases of the crossover design, respectively. These descriptive values help to visualize the treatment-by-period interaction discussed in the main text. Notably, Whitening Toothpaste and Liquid Soap showed markedly higher mean NBS values in T2 compared to T1, whereas Efferdent exhibited a substantial decrease in discoloration over time. These findings support the interpretation that treatment effects may differ by period and highlight the potential for carryover or compliance-related effects. No statistical comparisons are presented in this table.

Abbreviations: NBS = National Bureau of Standards; T1 = Period 1; T2 = Period 2.
