# Supplementary material for: Comparative evaluation of the effect of different cleaning agents on colour and surface roughness of Invisalign clear aligners: a cross-over randomized controlled trial
Source: BMC Oral Health. 2025 Nov 4;25:1745. doi: 10.1186/s12903-025-06928-w (PMC12584337; doi:10.1186/s12903-025-06928-w)
Supplement: Supplementary file 3 — Additional file 3. [file 12903_2025_6928_MOESM3_ESM.docx]

# Table S2 Mean color change (NBS) by arch for each treatment.

| Group Name | Upper | Lower |
| --- | --- | --- |
| Efferdent | 15.6 | 13.2 |
| Cleaning Crystals | 26.9 | 22.3 |
| Toothpaste | 26.1 | 22.7 |
| Liquid Soap | 15.1 | 19.8 |
| Whitening Toothpaste | 22.9 | 23.7 |

Note. This table presents the mean color change values (NBS units) by arch (upper and lower) for each cleaning agent. These values provide insight into whether discoloration differed between upper and lower aligners. Notably, Liquid Soap and Whitening Toothpaste showed greater discoloration in lower aligners, whereas Cleaning Crystals and Toothpaste showed higher values in upper aligners.

Abbreviations: NBS = National Bureau of Standards.
