# Supplementary material for: Comparative evaluation of the effect of different cleaning agents on colour and surface roughness of Invisalign clear aligners: a cross-over randomized controlled trial
Source: BMC Oral Health. 2025 Nov 4;25:1745. doi: 10.1186/s12903-025-06928-w (PMC12584337; doi:10.1186/s12903-025-06928-w)
Supplement: Supplementary file 4 — Additional file 4. [file 12903_2025_6928_MOESM4_ESM.docx]

**Table S3** Post hoc comparisons for cleaning agent × period × arch interaction

| **Comparison** | | | | | | | |  | | | | |
| --- | --- | --- | --- | --- | --- | --- | --- | --- | --- | --- | --- | --- |
| **Group**  **Name** | **Period** | | **Arch** | **vs** | **Group Name** | **Period** | **Arch** | **Difference** | **SE** | **t** | **df** | **p_bonferroni_** |
| Efferdent | | T1 | Upper | - | Cleaning Crystals | T1 | Upper | -15.62461 | 4.03 | -3.87735 | 180 | 0.028 |
| Efferdent | | T1 | Upper | - | Toothpaste | T1 | Upper | -15.93846 | 3.83 | -4.15967 | 218 | 0.009 |
| Efferdent | | T1 | Lower | - | Cleaning Crystals | T1 | Upper | -17.69294 | 4.03 | -4.39062 | 180 | 0.004 |
| Efferdent | | T1 | Lower | - | Toothpaste | T1 | Upper | -18.0068 | 3.83 | -4.69947 | 218 | <.001 |
| Efferdent | | T1 | Lower | - | Toothpaste | T1 | Lower | -15.89846 | 3.83 | -4.14923 | 218 | 0.009 |
| Efferdent | | T2 | Upper | - | Cleaning Crystals | T1 | Upper | -20.41865 | 3.74 | -5.4565 | 218 | <.001 |
| Efferdent | | T2 | Upper | - | Cleaning Crystals | T1 | Lower | -15.61615 | 3.74 | -4.17313 | 218 | 0.008 |
| Efferdent | | T2 | Upper | - | Toothpaste | T1 | Upper | -20.73251 | 4.06 | -5.11136 | 173 | <.001 |
| Efferdent | | T2 | Upper | - | Toothpaste | T1 | Lower | -18.62418 | 4.06 | -4.59158 | 173 | 0.002 |
| Efferdent | | T2 | Lower | - | Cleaning Crystals | T1 | Upper | -23.09365 | 3.74 | -6.17135 | 218 | <.001 |
| Efferdent | | T2 | Lower | - | Cleaning Crystals | T1 | Lower | -18.29115 | 3.74 | -4.88797 | 218 | <.001 |
| Efferdent | | T2 | Lower | - | Cleaning Crystals | T2 | Upper | -15.10863 | 3.98 | -3.7978 | 192 | 0.037 |
| Efferdent | | T2 | Lower | - | Toothpaste | T1 | Upper | -23.40751 | 4.06 | -5.77085 | 173 | <.001 |
| Efferdent | | T2 | Lower | - | Toothpaste | T1 | Lower | -21.29918 | 4.06 | -5.25107 | 173 | <.001 |
| Efferdent | | T2 | Lower | - | Toothpaste | T2 | Upper | -15.18662 | 3.83 | -3.96345 | 218 | 0.019 |
| Efferdent | | T2 | Lower | - | Liquid Soap | T2 | Lower | -15.1963 | 4.03 | -3.77106 | 180 | 0.042 |
| Efferdent | | T2 | Lower | - | Whitening Toothpaste | T2 | Lower | -17.28281 | 3.99 | -4.33251 | 188 | 0.005 |
| Cleaning Crystals | | T1 | Upper | - | Liquid Soap | T1 | Upper | 23.58917 | 3.87 | 6.0892 | 213 | <.001 |
| Cleaning Crystals | | T1 | Upper | - | Liquid Soap | T1 | Lower | 17.90917 | 3.87 | 4.62299 | 213 | 0.001 |
| Cleaning Crystals | | T1 | Upper | - | Whitening Toothpaste | T1 | Lower | 15.8273 | 3.92 | 4.03862 | 207 | 0.014 |
| Cleaning Crystals | | T1 | Lower | - | Liquid Soap | T1 | Upper | 18.78667 | 3.87 | 4.84951 | 213 | <.001 |
| Cleaning Crystals | | T2 | Upper | - | Liquid Soap | T1 | Upper | 15.60415 | 4.02 | 3.88421 | 188 | 0.027 |
| Toothpaste | | T1 | Upper | - | Liquid Soap | T1 | Upper | 23.90303 | 4.03 | 5.93169 | 180 | <.001 |
| Toothpaste | | T1 | Upper | - | Liquid Soap | T1 | Lower | 18.22303 | 4.03 | 4.52216 | 180 | 0.002 |
| Toothpaste | | T1 | Upper | - | Whitening Toothpaste | T1 | Upper | 14.82116 | 3.98 | 3.72554 | 192 | 0.049 |
| Toothpaste | | T1 | Upper | - | Whitening Toothpaste | T1 | Lower | 16.14116 | 3.98 | 4.05735 | 192 | 0.014 |
| Toothpaste | | T1 | Lower | - | Liquid Soap | T1 | Upper | 21.7947 | 4.03 | 5.40849 | 180 | <.001 |
| Toothpaste | | T1 | Lower | - | Liquid Soap | T1 | Lower | 16.1147 | 4.03 | 3.99896 | 180 | 0.018 |
| Toothpaste | | T2 | Upper | - | Liquid Soap | T1 | Upper | 15.68214 | 3.74 | 4.19076 | 218 | 0.008 |
| Liquid Soap | | T1 | Upper | - | Liquid Soap | T2 | Lower | -15.69182 | 4.03 | -3.89291 | 183 | 0.026 |
| Liquid Soap | | T1 | Upper | - | Whitening Toothpaste | T2 | Upper | -14.91333 | 3.8 | -3.92477 | 206 | 0.022 |
| Liquid Soap | | T1 | Upper | - | Whitening Toothpaste | T2 | Lower | -17.77833 | 3.8 | -4.67876 | 206 | <.001 |

Note. Linear mixed model post hoc comparisons evaluating colour change (NBS units) across combinations of cleaning agent, treatment period (T1 vs. T2), and dental arch (upper vs. lower). Each row shows the mean difference, standard error (SE), t-value, degrees of freedom (df), and Bonferroni-adjusted p-value.

*Only statistically significant comparisons (p < 0.05) are shown; non-significant comparisons were omitted.*
