# Supplementary material for: Comparative evaluation of the effect of different cleaning agents on colour and surface roughness of Invisalign clear aligners: a cross-over randomized controlled trial
Source: BMC Oral Health. 2025 Nov 4;25:1745. doi: 10.1186/s12903-025-06928-w (PMC12584337; doi:10.1186/s12903-025-06928-w)
Supplement: Supplementary file 5 — Additional file 5. [file 12903_2025_6928_MOESM5_ESM.docx]

**Table S4** Pairwise comparisons of cleaning agent × period × arch interaction on surface roughness

| **Comparison** | | | | | | |  | | | | |
| --- | --- | --- | --- | --- | --- | --- | --- | --- | --- | --- | --- |
| **Group Name** | **Period** | **Arch** | **vs** | **Group Name** | **Period** | **Arch** | **Difference** | **SE** | **t** | **df** | **p_bonferroni_** |
| **Efferdent** | **T1** | **Upper** | **-** | **Cleaning Crystals** | **T1** | **Upper** | **-0.08509** | **0.021** | **-3.9829** | **139** | **0.021** |
| **Efferdent** | **T1** | **Upper** | **-** | **Cleaning Crystals** | **T1** | **Lower** | **-0.09893** | **0.021** | **-4.6323** | **140** | **0.002** |
| **Efferdent** | **T1** | **Upper** | **-** | **Cleaning Crystals** | **T2** | **Upper** | **-0.0903** | **0.02** | **-4.4935** | **183** | **0.002** |
| **Efferdent** | **T1** | **Upper** | **-** | **Cleaning Crystals** | **T2** | **Lower** | **-0.0778** | **0.02** | **-3.8715** | **183** | **0.029** |
| **Efferdent** | **T1** | **Upper** | **-** | **Toothpaste** | **T2** | **Upper** | **-0.08474** | **0.021** | **-4.0287** | **132** | **0.018** |
| **Efferdent** | **T1** | **Upper** | **-** | **Toothpaste** | **T2** | **Lower** | **-0.0799** | **0.021** | **-3.7357** | **137** | **0.052** |
| **Efferdent** | **T1** | **Upper** | **-** | **Liquid Soap** | **T2** | **Upper** | **-0.08478** | **0.02** | **-4.2209** | **185** | **0.007** |
|  |  |  |  |  |  |  |  |  |  |  |  |

Note. This table reports significant post hoc comparisons from the linear mixed model evaluating the three-way interaction between cleaning agent, treatment period (T1 vs. T2), and arch (upper vs. lower). Only statistically significant comparisons are shown (p < 0.05, Bonferroni-adjusted).
